# Cytokine Based Immunotherapy for Cancer and Lymphoma: Biology, Challenges and Future Perspectives

**DOI:** 10.3389/fimmu.2022.872010

**Published:** 2022-04-20

**Authors:** Suheil Albert Atallah-Yunes, Michael J. Robertson

**Affiliations:** Department of Hematology and Medical Oncology, Indiana University School of Medicine, Indianapolis, IN, United States

**Keywords:** cytokines, immunotherapy, interleukins, cancer, lymphoma

## Abstract

Cytokines regulate both the innate and adaptive immune responses to cancer. Although antitumor activity has been seen for several cytokines in preclinical models, they have had limited success as single therapeutic agents in clinical trials of cancer immunotherapy. However, the possible combinations of cytokines with other immune therapeutics and the advancement in genetic engineering, synthetic biology and cellular and immune therapy has led to the revival of interest in cytokines as anticancer agents. This article will review several immunostimulatory cytokines with anticancer activity, focusing on the those that have been studied in treatment of lymphoma and highlighting recent advances of potential clinical relevance.

## Introduction

Cytokines are small glycoproteins and polypeptides that typically have a relatively short half-life and act in an autocrine and paracrine fashion. Cytokines mediate interactions between immune and nonimmune cells in the tumor microenvironment, which can either promote or inhibit the growth of cancer cells. Some cytokines, including interleukin-2 (IL-2), IL-12, IL-15, IL-18, IL-21, GM-CSF, CCL21 and type 1 interferons, have shown to have antitumor activity in preclinical studies (1, 2). Antitumor cytokine signaling could play a role in tumor antigen presentation, T-cell priming and activation, T cell infiltration and cancer cell death *via* stimulation of the adaptive and innate cell immunity (2, 3). Several cytokines, including IL-1B, IL-12, IL-18 and interferon (IFN)-γ, promote the differentiation of CD4 T cells into Th1 cells that can secrete cytokines, such as IL-2 and IFN-γ, that promote an antitumor response (4–7). Activation of natural killer (NK) cells by cytokines, including IL-2, IL-12, IL-15, IL-18, IFN-γ and CCL-5, can also augment antitumor immune responses. The cytokine antitumor activity seen in preclinical models led to the study of cytokines in clinical trials with GM-CSF, G-CSF, IL-2 and IFN-α being among the first studied (2). Cytokines have been explored in many solid and hematologic malignancies with the larger clinical trials mainly conducted in patients with melanoma, renal cell cancer, glioblastoma, breast cancer, lymphomas and leukemias (8). Despite the anticancer activity of numerous cytokines in preclinical models, only IL-2 and IFN-α showed sufficient clinical benefit as monotherapy in human clinical trials to warrant FDA approval. IL-2 is approved for treatment of advanced renal cell carcinoma (RCC) (9) and metastatic melanoma (10).IFN-α is approved for treatment of follicular lymphoma (11), hairy cell leukemia (12), AIDS-related Kaposi’s sarcoma (13), and melanoma (14). However, these cytokines have largely been supplanted in clinical practice by other immunotherapeutic targets, such as immune checkpoint inhibitors, with superior efficacy and more favorable toxicity profiles. Nevertheles, the possibility of combining cytokines with other immune therapies and the advancement in genetic engineering, synthetic biology and cellular and immune therapy have led to the revival of interest in cytokines as anticancer agents. This article will review several immunostimulatory cytokines with anticancer activity (**Table 1**), focusing on the those that have been studied in lymphoma (**Table 2**) and highlighting recent advances of potential clinical relevance.

**Table 1:** Cytokines investigated in Cancer Immunotherapy.

Cytokine Class	Examples used in clinical trials
Type I interferons	IFN-α2b
γ common chain receptor cytokines	IL-2, IL-15, IL-21
Heterodimeric cytokines	IL-12
IL-1 superfamily cytokines	IL-18

**Table 2:** Cytokine-based Immunotherapy for Lymphoma: Clinical Trial Results.

Investigators	Intervention	Lymphoma subtype and number of patients	Study phase	Results
Rook et al. (15)	Intralesional or subcutaneous (SC) IL-12 monotherapy	10 patients with cutaneous T cell lymphoma	Phase 1	responses occurred in 8/9 patients available for response assessment, including 2 CR
Duvic et al. (16)	IL-12 monotherapy	23 patients with early stage mycosis fungoides who received at least prior 3 antilymphoma therapy	Phase 2	73% had partial and minor responses while 22% had SD. 52% eventually progressed but some still achieved responses when continuing IL-12
Younes et al. (17)	Intravenous or subcutaneous IL-12	32 patients with NHL and 10 patients with HL who received a median of three prior antilymphoma therapies	Phase 2	6/29 patients (21%) with NHL had PR or CR while 10 patients (34%) had SD. FL patients had better responses and lower rate of progression than DLBCL patients (27% vs 64%). Median PFS for indolent NHL and aggressive NHL patients was 6 and 2 months respectively. No responses were seen in patients with HL but 5/10 patients (50%) had stable disease with median PFS of 2 months.
Ansell et al. (18)	Subcutaneous IL-12 + rituximab	43 patients with CD20 + NHL including indolent NHL, DLBCL and mantle cell lymphoma	Phase 1	Responses occurred in 29/43 patients (69%) with more responses in those who received higher doses of IL-12
Ansell et al. (19)	Subcutaneous IL-12 + rituximab or rituximab alone followed by subcutaneous IL-12 if no responses or progression with rituximab.	58 patients with relapsed B-cell NHL were randomized to receive rituximab + subcutaneous IL-12 (Arm A) or rituximab with subsequent treatment with IL-12 after documented nonresponse or progression after rituximab (Arm B)	Phase 2	Responses occurred in 11/30 patients (37%) in arm A and 13/25 patients (52%) in arm B. All of the responses in arm B occurred while patients received rituximab, and no responses occurred during treatment with subsequent IL-12.
Robertson et al. (20)	IL-18 + rituximab	19 patients with CD20+ NHL	Phase 1	5 patients achieved responses including 2 CR. ORR was 62% in patients having indolent NHL
Robertson et al. (21)	IL-18 + ofatumumab post-PBSCT	9 patients with NHL including 7 with DLBCL	Phase 1	The 7 patients who were not taken off study early for lymphoma progression remained alive without evidence of active lymphoma at a median of 3.5 years post PBSCT. They remained progression free for more than 2.4 years post PBSCT
Timmerman et al. (22)	IL-21 + rituximab	21 patients with indolent R/R NHL including 9 patients with FL and 1 patient with marginal zone lymphoma.	Phase 1	Of 19 patients with evaluable responses, objective response occurred in 8 patients including 3 CR/CRu and 5 PR. Third of the patients who had rituximab resistant disease responded

## Immunostimulatory Cytokines for Cancer Immunotherapy

### Biology of IFN-α

Interferons are cytokines that are produced by malignant cells and dendritic cells. They are classified based on antigenic specificity into IFN-α, β and γ. IFN-α is a type 1 interferon and has been most extensively studied in anticancer therapy. The anticancer activity of IFN-α is likely due to its effect on multiple immune cells. It enhances proliferation and cytotoxicity of CD8 T cells. It also enhances cytotoxicity of NK cells and their expansion by stimulating IL-15 production. It plays a role in activation of the STING pathway post cytosolic DNA which plays a role in activation of Batf3+ dendritic cells, central to antigen presentation and hence to T cell effector functions. It may also upregulate the expression of MHC class I molecules on tumor cells. It also enhances the expression of PD-1 and PDL1 ligand on T-cell and neoplastic cells respectively, hence there is an interest in studying IFN-α in combination with immune checkpoint inhibitors (23). In preclinical models of lymphoma IFN-α was shown to have direct antitumor effects on neoplastic B cells by inducing apoptosis, inhibiting proliferation and cell cycle progression and promoting terminal differentiation in cancer cells (24, 25).

#### IFN-α-Based Immunotherapy for Cancer

Phase II trials involving IFN-α were conducted by the National Cancer Institute in non-Hodgkin lymphoma (NHL) patients. Conflicting results were seen in regards to the impact of IFN-α induction monotherapy and maintenance, and when combined with chemotherapy, on survival in NHL patients. IFN-α was used in treating low grade indolent NHLs where it showed some activity, however complete response (CR) and overall response rates were only 10% and 48% respectively (26–28). The introduction of rituximab in the late 1990s, led to better results when it was combined with interferon due to enhanced ADCC (28). IFN-α may also have a role in the treatment of myeloproliferative diseases (29). Several strategies to overcome the narrow therapeutic index of IFN-α include delivering IFN-α into tumor cells *via* immunocytokines and genetically engineered dendritic cells expressing vectors encoding IFN-α.

### Biology of IL-2

IL-2 is one of the first cytokines to have been studied in anticancer treatment. IL-2 is a 4 alpha helix cytokine that has a major role in innate and adaptive immune responses. The IL-2 receptor has three subunits: IL-2Rα (CD25), IL-2Rβ (CD122), and the γ common chain (CD132). CD132 is also a component of the receptors for IL-4, IL-7, IL-9, IL-15, and IL-21. IL-2Rα binds IL-2 with low affinity and the IL-2Rβγc heterodimer binds IL-2 with intermediate affinity. The high affinity receptor for IL-2 is the IL-2Rαβγc heterotrimer (30). IL-2Rαβγc is constitutively expressed by CD4+ regulatory T cells (Tregs) and CD565^bright^ NK cells and is transiently expressed on activated CD4+ and CD8+ T cells. The intermediate affinity IL-2Rβγc is constitutively expressed by CD56^dim^ NK cells. IL-2 mediates antitumor immunity by promoting the proliferation and differentiation of activated CD8+ T cells into cytotoxic T lymphocytes (CTL) and by stimulating cytotoxicity and cytokine production of CTL and NK cells. However, IL-2 can also stimulate the proliferation and effector functions of Tregs, resulting in immunosuppressive effects that are not desirable in anticancer therapy (31–34).

#### IL-2-Based Immunotherapy Therapy for Lymphoma

Several studies established that continuous low dose of IL-2 results in expansion of NK cells while pulse intermediate dose IL-2 increases cytotoxic activity of NK cells (35–38). Synergistic activity against NHL was seen in mouse model when daily low dose IL-2 was administered with intermittent pulse intermediate dose IL2 and rituximab, likely due to enhanced ADCC mediated by NK cells. Although some early phase 1 studies showed promising results for the combination of rituximab and IL-2 (39, 40), other studies showed no significant clinical benefit (41, 42). Most of these studies used low dose IL-2, which could have preferentially expanded Tregs. The IL-2-based immunocytokines that showed positive results in preclinical lymphoma studies include L19-IL2 in NHL, HI-Leu 16-IL2 in lymphoma and HRS3scFv-IL12-Fc-IL12 in Hodgkin lymphoma. Diphteria toxin-IL-2 fused proteins showed promising results in a phase III trials of cutaneous T cell lymphoma (CTCL) patients (43). Currently, a phase II trial is ongoing for evaluating a similar diphtheria toxin-IL-2 fused protein with high bioavailability in patients with refractory/relapsed CTCL and peripheral T cell lymphomas (44).

#### Challenges and Future Directions for IL-2-Based Cancer Immunotherapy

High dose IL-2 is approved by the FDA for treatment of metastatic RCC and metastatic melanoma. However due to the various challenges with high dose IL-2 monotherapy including its substantial toxicity and modest efficacy, it is infrequently used and has largely been replaced by other immunotherapeutic agents (45). Low dose IL-2 could preferentially expand Tregs over the activation of NK and CD8 T cells (30). The high doses of IL-2 required for immune stimulation of NK and CD8 T cells is associated with serious side effects such as hypotension, organ failure, cytopenias and vascular leak syndrome (46). To improve the pharmacokinetics and pharmacodynamics of IL-2 and reduce its systemic toxicity several strategies have been pursued. Immunocytokines, in which IL-2 is linked to an antibody that targets tumor associated-antigens, have been efficacious in preclinical models and are currently being used in IL-2 based therapy trials (45). IL-2-based immunocytokines have been tested in combination with other cytokines which enhance the activation of NK cells (47). Combining IL-2 immunocytokines with other immunocytokines, chemotherapeutics, and immunotherapeutic agents such as immune checkpoint inhibitors will likely be implemented in future IL-2-based immunotherapies (45, 48). The administration of intratumoral IL-2 may decrease systemic toxicity (45, 48). Adjusting the design of manufactured IL-2/IL-2 immunocytokines to preferentially stimulate NK and CD8 T cells over the expansion of Tregs is one of approaches to optimize IL-2-based therapies. The structure of IL-2 cytokine/immunocytokines have been engineered to have higher affinity for the IL-2Rβγc heterodimer and diminished binding to the IL-2Rα on Tregs. IL-2 has also been successfully combined with an IL-2 antibody that masks the IL-2Rα binding site of IL-2, thus abolishing binding of engineered IL-2 to Tregs. Pegylation of IL-2-based immunocytokines can increase their half life and also block IL-2 from binding to IL-2Rα (49–51). Novel IL-2 fusion proteins have been designed to enhance the activity and proliferation of NK cells and have shown promising results in preclinical models. The OCMP-mutIL-2 fusion protein binds with high affinity to NKG2D on NK cells and contains mutations that confer preferential binding to IL-2Rβγc rather than IL2Rα (52, 53). Adjusting the structure of the IL-2-based therapies can also alleviate the risk of toxicities such as vascular leak syndrome.

### Biology of IL-12

IL-12 is a heterodimeric cytokine that is secreted by antigen presenting cells (APCs), including dendritic cells and cells of monocyte/macrophage lineage, in response to pathogen-associated molecular patterns, damage-associated molecular patterns, cytokine stimulation and direct immune cell–cell contact (1, 54). IL-12 binds with high affinity to the IL-12Rβ1/IL-12Rβ2 heterodimer that is constitutively expressed by NK cells and after activation by T cells and B cells. Binding of IL-12 to its receptor leads to recruitment and activation of JAK2 and TYK2 with subsequent activation of STAT4 (54). IL-12 has been used in cancer immunotherapy based on its anti-tumor activity in preclinical models mediated by various effects on the adaptive and the innate immune system and bridging them together (54). IL-12 is critical for the production of IFN-γ by NK and T cells (55). IL-12 promotes the differentiation of naïve CD4 Th0 cells into Th1 cells (56) and the CD8 T cells into CTL. It also augments the cytolytic activity and the growth of activated T cells and NK cells (57). IL-12 enhances NK mediated ADCC (58), B cell survival and IgG production. IL-12 also has antiangiogenic effects by enhancing the production of monokine induced by IFN-γ (MIG; CXCL9) and IFN-γ-induced protein 10 (IP-10;CXCL10) (59). IL-12 also exerts antitumor effects through regulation of peritumoral extracellular matrix, tumor stroma, and enhancing the processing and expression of MHC class I molecules (54).

#### IL-12-Based Immunotherapy for Lymphoma

The decrease in function of Th1 cells and underproduction of IFN-γ in the tumor environment of CTCL, makes IL-12 an attractive therapeutic option for this lymphoma subtype, especially in as much as cutaneous lesions could be easily accessed (54, 60). In a study of 10 patients with CTCL who received biweekly intralesional or subcutaneous (SC) injections of IL-12 monotherapy, 9 patients were available for response assessment at safety analysis time, responses occurred in 8/9 patients including 2 CR. Side effects were short lived (15). SC IL-12 monotherapy was also evaluated in a phase II trial of 23 patients with early stage mycosis fungoides whom received at least 3 prior antilymphoma therapies, 10/23 patients received IL-12 injections for at least 6 months and continued for 2 years. 73% had partial and minor responses while 22% had stable disease. However, 52% eventually progressed but some still achieved responses when continuing IL-12. 5 patients stopped therapy due to side effects and 1 patient died with hemolytic anemia that was likely attributed to the drug (16). A recent phase 2 study (NCT02542124) combining IL-12 with low dose total skin electron beam therapy has been initiated with the rationale that IL-12 may also decrease radiation toxicity.

The first study of IL-12 monotherapy in NHL and Hodgkin lymphoma (HL) patients was reported by Younes et al. (17) in which 32 patients with NHL and 10 patients with HL who received a median of three prior antilymphoma therapies were enrolled. NHL patients had diffuse large B-cell lymphoma (DLBCL) and grade 1-2 follicular lymphoma (FL). 11 patients received 250 ng/kg of intravenous (IV) IL-12 daily for 5 days every 3 weeks (preceded by an initial test dose of 250 ng/kg) and 31 patients received twice-weekly IL-12 SC injections at 500 ng/kg (in case of toxicity, the dosage was reduced to 300 ng/kg). 39 patients (93%) were present for response assessment. 6/29 patients (21%) with NHL had partial remission (PR) or CR while 10 patients (34%) had stable disease. FL patients had better responses and lower rate of progression than DLBCL patients (27% vs 64%). Median progression free survival (PFS) for indolent NHL and aggressive NHL patients was 6 and 2 months respectively. Overall responses in those who received IV and SC IL-12 were 40% and 7% respectively. All responders had low burden disease. IL-12 did not affect the CD4 T cell count but the mean CD8 T cell count was significantly increased. Levels of VEGF and bFGF (reflecting angiogenesis) were either decreased or stable after IL-12 injections in most patients who responded, and were stable or increased in most patients who did not respond or progressed. No responses were seen in patients with HL but 5/10 patients (50%) had stable disease with median PFS of 2 months. All HL patients received SC IL-12 and it is possible this could have influenced the results. IL-12 was tolerable in most patients with 95% developing fevers that mostly responding to acetaminophen. Fatigue, malaise and arthralgia were commonly reported and were more common in those who received IV IL-12. 9 patients required dose reductions including 3 patients who developed grade 3 hepatotoxicity and all received SC IL-12. All hepatotoxicities were grade 1 and 2. Treatment was prematurely stopped in 4 patients (17). In a phase 1 study of a group with heterogenous types of lymphomas, IL-12 was given SC twice weekly at doses 500 and 300 ng/kg or lower along with rituximab with the aim to enhance NK mediated ADCC. PR and CR were seen in 69% with more responses in those who received higher doses of IL-12 (18). However, in the phase 2 study, comparable response rates were seen with rituximab monotherapy and combination of rituximab and IL-12 (19). A phase II study has been initiated to evaluate the combination of IL-12 with salvage R-ICE or R-DHAP in patients with relapsed/refractory aggressive B-cell NHL with the rationale that IL-12 may enhance responses but also decrease chemotherapy toxicity (NCT02544724).

The safety of using IV IL-12 after autologous peripheral blood stem cell transplantation (PBSCT) was established in a phase 1 trial by Robertson et al. in which 12 patients including 10 with hematologic malignancies (8 NHL, 2 HL, 2 plasma cell myeloma) with very high risk of relapse received three different initial doses of IV IL-12. Time to progression ranged from 10.5 to 50.8 months with 3/8 of the NHL patients and 1/2 of HL patients, did not progress during the observation period (median of 32.4 months after initiating IL-12 therapy) (61). An obstacle to optimal IL-12-based immunotherapy after PBSCT is the profound, acquired STAT4 deficiency that can occur in this setting (62).

#### Challenges and Future Directions for IL-12-Based Cancer Immunotherapy

Significant systemic toxicities have been reported in studies with IL-12 that are mainly due to increased production of IFN-γ, TNF, IP-10 and MIG. Reported adverse events and toxicities in clinical trials include fatigue, fever, arthralgia, headaches and malaise (63–67). Erythropoietin analogues and G-CSF were successful in reducing anemia and bone marrow suppression respectively (54). It seems that systemic toxicity may be reduced if IL-12 is delivered locally into the tumor bed and is being implemented in current IL-12 clinical trials (54).

The anticancer effect of IL-12 in preclinical models was enhanced by combining it with chemotherapeutics, other cytokines, antibodies, antiangiogenic agents, radiotherapy, adoptive therapy, and tumor vaccines. For example, in a preclinical Burkitt lymphoma model, the anticancer effect of IL-12 was potentiated by the addition of vasostatin. Chemotherapeutic agents could augment the anticancer activity by increasing the release and presentation of tumor antigens. However, some chemotherapeutic agents could suppress the immune response by inhibiting immune effector cells (62).

Despite the anticancer activity seen in preclinical models, IL-12 monotherapy did not yield the same results in human clinical trials. One possible explanation for this discrepancy is the negative feedback and immunoregulatory effect that could result from increased IL-10 and TIM3 production that could lead to an increase in IFN-γ levels and increase PDL1 expression on cancer cells (54). Also, the human tumor environment may be more heterogenous than preclinical models with more immunosuppressive effect and tumor escape mechanisms.

Delivering IL-12 regionally or locally into the tumor environment may overcome its narrow therapeutic index by enhancing efficacy and decreasing systemic toxicity. With the advancement in genetic engineering techniques and adoptive cell therapies, IL-12 is regaining attention (68). In recent cancer experiments and early studies, IL-12 has been genetically delivered *via* engineered viruses or nonviral vectors such as IL-12 expressing plasmids (69–71). IL-12 plasmids are being studied in CTCL (54). Adoptive immunotherapy with the utilization of genetically modified IL-12 expressing lymphocytes have also been implemented (72). The idea of delivering CAR T cells transduced with inducible IL-12 is also being considered (73). IL-12 has also been combined with cancer vaccines including dendritic cell-tumor cell fusion vaccines (74). IL-12-based Immunocytokines in which IL-12 is attached to monoclonal antibodies that target specific tumor antigens may also help decrease systemic toxicity and improve efficacy of IL-12.

### Biology of IL-15

IL-15 is a four alpha helix cytokine (75). The receptor for IL-15 is a heterotrimer that includes IL-15Rα (CD215), which is specific to IL-15, CD122 (β subunit common to IL-2 and IL-15) and the γc chain (CD132) (76). IL-15 is produced by APCs (macrophages, monocytes, and dendritic cells) as well as several other cell types. After IL-15 protein is translated from mRNA, it binds with high affinity to intracellular IL-15Rα and the IL-15/IL-15α complex is transported to the cell surface of the producing cells. The IL-15-IL-15Rα complex is transpresented to the IL2/IL-15Rβγc heterodimer expressed on NK and CD8 T cells (75, 77–79). The main immunostimulatory effects of IL-15 that may lead to its antitumor activity include enhancing the proliferation and survival of T cells, proliferation and differentiation of NK cells, and differentiation of CTL. IL-15 can cause prolonged expansion and activation of both NK cells and CD8 T cells (78, 80, 81). Despite sharing some biologic effects with IL-2, the use of IL-15 in anticancer therapy has potential advantages compared to IL-2. Potential advantages of IL-15 are that it causes less expansion of Tregs and less activation-induced death of effector T cells compared to IL-2. IL-15 also does not appear to cause major capillary leakage that can be a serious toxicity of IL-2 (82, 83).

#### Challenges and Future Directions for IL-15-Based Cancer Immunotherapy

Despite the potent antitumor activity of IL-15 seen in some preclinical models, clinical results have not been as impressive, with issues that include a short half-life, relatively modest bioactivity *in vivo*, and reliance on transpresentation. Several approaches have been taken to overcome these challenges. Constructing superagonist complexes by combining IL-15 with various forms of IL-15Rα led to a superior antitumor activity than IL-15 monotherapy. Administration of a superagonist is thought to mimic transpresentation of IL-15 by IL-15Rα to effector cells expressing the IL-2/IL-15βγc heterodimer and thus enhancing IL-15 bioactivity (77, 79). Novel IL-15-based therapies have combined it in preclinical studies with other immunotherapeutic agents, such as monoclonal antibodies (rituximab, alemtuzumab), CD40 agents, cancer vaccines, and other cytokines (IL-12, IL-18, and IL-21) (82, 84). When these preclinical model approaches translated to human clinical trials, tumor responses have been disappointing, perhaps due to the immunosuppressive tumor microenvironment. Thus, combining IL-15-based therapies with immune checkpoint inhibitors that target the immunosuppressive environment is rational (82). IL-15-based therapy with dendritic vaccines and oncolytic viruses expressing IL-15 or IL-15- IL-15Rα are also being investigated in preclinical studies (82, 85). Toxicities and adverse events that were observed with recombinant IL-15 include fever, hypotension, appetite changes, weight loss, diarrhea, rash, transient grade 3–4 neutropenia and anemia. These adverse events were less severe when IL-15 was given SC and intermittently. A concern with the use of IL-15 arises from its potent stimulatory effect on NK cells and CD8 T cells, which could lead to autoimmune toxicities (78). Future studies are needed to determine the optimal administration route and dosage for IL-15- based therapy to minimize toxicity and enhance its bioactivity. Most of the ongoing/recruiting early phase clinical trials of IL-15 based therapy in lymphoma patients are using IL-15 super agonists and in combination with other anticancer therapeutics due to the challenges with IL-15 monotherapy mentioned before. Phase 1 trial has been initiated to evaluate the safety and efficacy of the IL-15 superagonist ALT -803 in combination with rituximab in patients with relapsed/refractory indolent NHL who received at least 1 prior rituximab treatment (NCT02384954).

### Biology of IL-18

IL-18 is an immunostimulatory cytokine that regulates both innate and adaptive immune responses. IL-18, a member of the IL-1 superfamily of cytokines, is produced by several cell types, including macrophages, dendritic cells, and epithelial cells. IL-18 binds to a receptor complex composed of at least two subunits, IL-18Rα (IL-1Rrp1) and IL-18Rβ (AcPL). Preclinical models and phase 1 trials established efficacy and biological activity of IL-18 against solid tumors and lymphoma (20, 86, 87). IL-18 could promote antitumor responses by stimulating production of IFN-γ, activating NK cells and monocytes/macrophages and enhancing their ADCC. Increased production of IFN-γ by IL-18 leads to increased production of CXCL9 (MIG) and CXCL10 (IP-10) that exert an antiangiogenic effect. IL-18 could also enhance the differentiation of Th1 cells and facilitate the priming of effector cells including helper NK cells into tumor site (20). Despite the biological activity of IL-18 seen in early studies, IL-18 monotherapy had limited efficacy in cancer patients (86–88). Thus, several approaches are being implemented in studies to enhance the efficacy of IL-18 such as combining it with other therapeutics.

#### IL-18-Based Immunotherapy for Lymphoma

IL-18 showed *in vitro* and *in vivo* synergistic activity when combined with the CD20 monoclonal antibody rituximab in a preclinical study (89). This led to a phase 1 study in which 19 patients with CD20+ NHL received rituximab and escalating doses of human recombinant IL-18. Eleven patients had rituximab-refractory disease. 5 patients achieved responses including 2 CR. The response rate was 62% for the 8 patients with indolent lymphoma who received doses of IL-18 that were associated with optimal biologic responses as defined by ancillary biomarker studies. Overall response rates were similar in patients with rituximab-refractory and rituximab-sensitive disease. The combination was deemed to be safe with a similar safety profile to IL-18 monotherapy. The objective responses are likely attributable to enhanced ADCC mediated by NK cells and monocyte/macrophages against rituximab-sensitized lymphoma cells (20). Although rituximab has been combined in clinical studies with other cytokines such as IL-2, IL-12 and GM-CSF, IL-18 has the advantage of enhancing the ADCC mediated by both NK cells and macrophages/monocytes. IL-2 and IL-12 enhance preferentially NK cell-mediated ADCC and GM-CSF preferentially enhances ADCC mediated by macrophages/monocytes (20). Ofatumumab is monoclonal antibody that binds to a different epitope of CD20 than that recognized by rituximab. Preclinical studies showed that IL-18 plus ofatumumab was more effective than IL-18 plus rituximab in a lymphoma xenograft model. IL-18-based immunotherapy might circumvent the acquired STAT4 deficiency that impairs IL-12-based treatment after PBSCT. A phase 1 study showed that the combination of ofatumumab with IL-18 in 9 patients with NHL (7 DLBCL) post high dose chemotherapy and PBSCT is feasible and tolerable with no dose limiting toxicities. Although efficacy could not be determined from this phase 1 study, the seven patients who were not taken off study early for lymphoma progression remained alive without evidence of active lymphoma at a median of 3.5 years post PBSCT. All of these patients have remained progression free for more than 2.4 years post PBSCT (21). Other approaches include utilizing IL-18 secreting CAR T cells and IL-18-based adoptive transfer (such as IL-12/IL-15/IL18 preactivated NK cells) (90, 91) with ongoing clinical trials as promising results were seen in early clinical trials of AML patients (92, 93).

#### Challenges and Future Directions for IL-18-Based Cancer Immunotherapy

IL-18 has a very favorable toxicity profile but has had limited efficacy as a monotherapy. IL-18 binding protein (IL-18BP) is a secreted antagonist that binds to IL-18 with high affinity and neutralizes its biological activity. IFN-γ induced by IL-18 may exert a negative feedback loop by increasing the expression of IL-18BP (91). Zhou et al. developed a form of IL-18 that is resistant to inhibition by IL-18BP and showed a potent antitumor responses in mice (94). IFN-γ induced by IL-18 could also stimulate upregulation of PD-L1 on tumors cells, providing a rationale for combining IL-18 and PD-1 checkpoint inhibitors in cancer immunotherapy. Other approaches to enhance the antitumor activity of IL-18 include combining it with chemotherapy and other immune therapeutics (monoclonal antibodies,other cytokines, cancer vaccines) (91). IL-18 secreting CAR T cells and IL-18-IL2 fusion proteins are under investigation.

Complicating the development of IL-18-based cancer immunotherapy are preclinical studies showing the IL-18 can promote tumor invasiveness and progression in some experiments. It is possible that IL-18 levels may have an impact on tumor regression/growth as administration of low dose IL-18 in murine melanoma model promoted metastasis due to suppressed mature NK cell number, while high doses of IL-18 that resulted in IL-18 serum levels > 1 ng/mL inhibited tumor growth without a decrease in mature NK cell number (95). The lowest doses of hIL-18 given to lymphoma patients resulted in IL-18 plasma levels of > 10 ng/mL and biomarker studies have shown *in vivo* activation rather than suppression of NK cells during IL-18-based immunotherapy (20, 87). Further studies are needed to determine the precise conditions under which IL-18 promotes or inhibits tumor growth versus regression. IL-18BP-based therapy may be a potential therapeutic modality for clinical scenarios where IL-18 can promote tumor growth.

### Biology of IL-21

IL-21 is a 4 helix bundle cytokine that binds to the IL-21R (CD360)/γc (CD132) heterodimeric receptor complex. IL-21 is mainly secreted by T follicular helper cells, Th17 cells and NK T cells. IL-21 stimulates innate and adaptive immune responses and showed antitumor activity in preclinical models. IL-21 enhances the proliferation of CD4 T cells and the proliferation and cytotoxic activity of CD8 T cells. IL-21 suppressed Treg cells *in vitro* and together with IL-16, IL-21 can enhance the differentiation of CD4 T cells into TH17 cells. IL-21 enhances maturation and cytotoxic activity of NK cells. ADCC activity of NK cells can be enhanced by IL-21 reflected by increased CD16 expression. At low doses, IL-21 enhances proliferation of NK cells while at high doses and in the presence of IL-2 and IL-15, IL-21 decreases the proliferation of NK cells (96). The effect of IL-21 on B-cells is variable and is influenced by the type and differentiation stage of the B lymphocyte which will be discussed in the next section. Despite the antitumor activity of IL-21 in preclinical models of solid and hematologic malignancies, this was not met with the same success when taken into human clinical trials. Current approaches to enhance the antitumor activity of IL-21 include combining it with other cytokines and monoclonal antibodies and co-administration with adoptively-transferred cells and tumor cell vaccines (96).

#### IL-21 Based Immunotherapy for Lymphoma

The effect of IL-21 on B-cells is influenced by the stage differentiation of the B-lymphocyte. IL-21 can either enhance the proliferation or apoptosis of the B cells. Given that IL-21 has an indirect antitumor activity by enhancing the cytotoxic activity and proliferation of CD8 T cells and NK cells and that it can promote apoptosis of the B-cell at certain stages by upregulation of pro-apoptotic proteins such as Bim and downregulation of anti-apoptotic proteins, there has been an interest in studying IL-21 in the treatment of lymphoma. IL-21 led to complete regression of DLBCL in xenograft models and extended their survival by promoting apoptosis of B-cells (96, 97). Glebert et al. showed that IL-21 had antiproliferative effect on 2 mantle cell lymphoma (MCL) cell lines with increased pro-apoptotic proteins and decreased anti-apoptotic protein (98). IL-21 combined with rituximab was evaluated in a phase 1 clinical trial involving 21 patients with indolent R/R NHL including 9 patients with FL and 1 patient with marginal zone lymphoma. Of 19 patients with evaluable responses, objective response occurred in 8 patients including 3 CR/CRu and 5 PR. A third of the patients who had rituximab-resistant disease responded (22). Contrarily, clinical studies showed that IL-21 can promote the proliferation of Hodgkin lymphoma (99), EBV transformed lymphomas (100) and ALK-positive anaplastic large cell lymphoma (101) suggesting that blocking the effect of IL-21 may be a potential therapeutic approaches in these lymphoma subtypes.

## Conclusion

Despite promising antitumor activity of several cytokines in preclinical models, cytokines as monotherapy have had limited success when tested in clinical trials. Indeed, only IFN-α and IL-2 are currently approved for treatment of cancer. However, advances in genetic engineering, synthetic biology, and cellular and immune therapy have led to a renewal of interest in cytokines for cancer immunotherapy. Combinations of cytokines with monoclonal antibodies to tumor antigens, checkpoint inhibitors, vaccines, and cell-based therapies are being investigated in clinical trials (**Table 3**). Moreover, autologous and/or allogeneic T cell products have been designed to express immunostimulatory cytokines in addition to CAR that direct them to tumor-associated antigens (102–104). A phase I clinical trial of ST-067, a recombinant human IL-18 engineered to abolish binding and neutralization of its activity by IL-18BP, has been initiated. Innovative approaches to cytokine-based cancer immunotherapy are being actively pursued and, it is to be hoped, will improve outcomes for patients with lymphoma and other malignancies.

**Table 3:** Cytokine-based Immunotherapy for Cancer: Clinical Trials.

Cytokine	Other Agents	Tumor types	ClinicalTrials.gov identifier
IL-2	Nivolumab	Melanoma, RCC	NCT03991130
IL-2	Pembrolizumab	RCC	NCT02964078
IL-2	Nivolumab; ipilimumab	Melanoma	NCT04562129
IL-2	Enoblituzumab	Ovarian cancer	NCT04630769
IL-2	TASO-001	Solid tumors	NCT04862767
IL-12	DC/tumor vaccine	Glioma	NCT04388033
IL-12	Cetuximab	Head and neck cancer	NCT01468896
IL-12	Pembrolizumab	Solid tumors	NCT03030378
IL-15	Mogamulizumab	CTCL	NCT04185220
IL-15	Nivolumab; ipilimumab	Solid tumors	NCT03388632
IL-15	Avelumab	PTCL, CTCL	NCT03905135

## Author Contributions

All authors listed have made a substantial, direct, and intellectual contribution to the work, and approved it for publication.

## Funding

The research of MJR is supported by gift from Eugene and Nancy Bate.

## Conflict of Interest

The authors declare that the research was conducted in the absence of any commercial or financial relationships that could be construed as a potential conflict of interest.

## Publisher’s Note

All claims expressed in this article are solely those of the authors and do not necessarily represent those of their affiliated organizations, or those of the publisher, the editors and the reviewers. Any product that may be evaluated in this article, or claim that may be made by its manufacturer, is not guaranteed or endorsed by the publisher.
